# Impact of genetic modulation of SULT1A enzymes on DNA adduct formation by aristolochic acids and 3-nitrobenzanthrone

**DOI:** 10.1007/s00204-016-1808-6

**Published:** 2016-08-24

**Authors:** Volker M. Arlt, Walter Meinl, Simone Florian, Eszter Nagy, Frantisek Barta, Marlies Thomann, Iveta Mrizova, Annette M. Krais, Maggie Liu, Meirion Richards, Amin Mirza, Klaus Kopka, David H. Phillips, Hansruedi Glatt, Marie Stiborova, Heinz H. Schmeiser

**Affiliations:** 1 Analytical and Environmental Sciences Division, MRC-PHE Centre for Environment and Health, King’s College London, Franklin-Wilkins Building, 150 Stamford Street, London, SE1 9NH UK; 2 Department of Nutritional Toxicology, German Institute of Human Nutrition (DIfE) Potsdam-Rehbrücke, 14558 Nuthetal, Germany; 3 Department of Biochemistry, Faculty of Science, Charles University, Albertov 2030, 12840 Prague 2, Czech Republic; 4 Division of Occupational and Environmental Medicine, Lund University, 221 85 Lund, Sweden; 5 Division of Cancer Therapeutics, Institute of Cancer Research, Sutton, Surrey, SM2 5NG UK; 6 Division of Radiopharmaceutical Chemistry, German Cancer Research Center (DKFZ), Im Neuenheimer Feld 280, 69120 Heidelberg, Germany; 7 Department of Food Safety, Federal Institute for Risk Assessment (BfR), 10589 Berlin, Germany

**Keywords:** Aristolochic acid nephropathy, Sulfotransferase 1A1, Carcinogen metabolism, DNA adducts, Balkan endemic nephropathy, 3-Nitrobenzanthrone

## Abstract

Exposure to aristolochic acid (AA) causes aristolochic acid nephropathy (AAN) and Balkan endemic nephropathy (BEN). Conflicting results have been found for the role of human sulfotransferase 1A1 (SULT1A1) contributing to the metabolic activation of aristolochic acid I (AAI) in vitro. We evaluated the role of human SULT1A1 in AA bioactivation in vivo after treatment of transgenic mice carrying a functional human *SULT1A1*-*SULT1A2* gene cluster (i.e. *hSULT1A1/2* mice) and *Sult1a1(−/−)* mice with AAI and aristolochic acid II (AAII). Both compounds formed characteristic DNA adducts in the intact mouse and in cytosolic incubations in vitro. However, we did not find differences in AAI-/AAII-DNA adduct levels between *hSULT1A1/2* and wild-type (WT) mice in all tissues analysed including kidney and liver despite strong enhancement of sulfotransferase activity in both kidney and liver of *hSULT1A1/2* mice relative to WT, kidney and liver being major organs involved in AA metabolism. In contrast, DNA adduct formation was strongly increased in *hSULT1A1/2* mice compared to WT after treatment with 3-nitrobenzanthrone (3-NBA), another carcinogenic aromatic nitro compound where human SULT1A1/2 is known to contribute to genotoxicity. We found no differences in AAI-/AAII-DNA adduct formation in *Sult1a1(−/−)* and WT mice in vivo. Using renal and hepatic cytosolic fractions of *hSULT1A1/2*, *Sult1a1(−/−)* and WT mice, we investigated AAI-DNA adduct formation in vitro but failed to find a contribution of human SULT1A1/2 or murine Sult1a1 to AAI bioactivation. Our results indicate that sulfo-conjugation catalysed by human SULT1A1 does not play a role in the activation pathways of AAI and AAII in vivo, but is important in 3-NBA bioactivation.

## Introduction

**Fig. 1:**
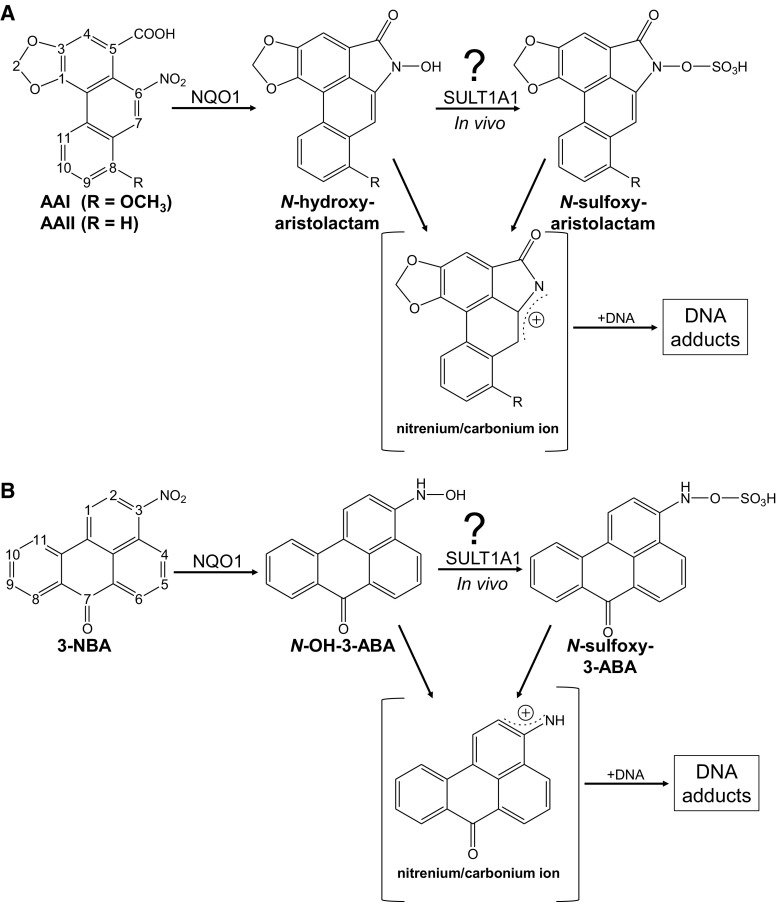
Proposed pathways of bioactivation and DNA adduct formation by AA (**a**) and 3-NBA (**b**). See *text* for details

Aristolochic acid (AA), the natural extract of *Aristolochia* plants consists of structurally related nitrophenanthrene carboxylic acids, the major components being aristolochic acid I (AAI) and aristolochic acid II (AAII) (Fig. 1a) (Arlt et al. 2002c). AA is found in all parts of plants of both the *Aristolochia* and *Asarum* genera of the family Aristolochiaceae. *Aristolochia* herbs have been used for remedies throughout the world since antiquity and they remain in use today, particularly in Chinese herbal medicine (Schmeiser et al. 2009).

In the 1970s the anti-inflammatory properties of AA prompted the production of pharmaceutical preparations until it was shown that AA is a strong carcinogen in rats (Mengs et al. 1982). Subsequently, AA was found to be a genotoxic mutagen and all pharmaceutical preparations containing AA were withdrawn from the market first in Germany and later in many other countries. In 2012 AA was classified by the International Agency for Research on Cancer (IARC) as carcinogenic to humans (Group 1) acting by a genotoxic mechanism. Today there is compelling evidence that human exposure to AA leads to chronic renal disease and upper urinary tract cancer known as aristolochic acid nephropathy (AAN) (Nortier et al. 2000), which is now recognised as a global disease (Gokmen et al. 2013).

The metabolism of AA has been studied in several species demonstrating that the major metabolites found in urine and faeces are the aristolactams I and II (Chan et al. 2006; Krumbiegel et al. 1987), produced by exhaustive six-electron reduction of the nitro group. Other minor metabolites are formed through *O*-demethylation of AAI to 8-hydroxyaristolochic acid I (AAIa) and through denitration. Further reduction of AAIa leads to aristolactam Ia. The only metabolites identified in humans so far are the aristolactams I and II found in urine (Krumbiegel et al. 1987).

Many studies have shown that AAI and AAII are generally bioactivated by reduction of the nitro group (Fig. 1a) (Stiborova et al. 2013, 2014a). Several human enzymes capable of activating AA by nitroreduction have been identified and include cytosolic NAD(P)H:quinone oxidoreductase (NQO1) (Stiborova et al. 2002, 2003), and microsomal enzymes including cytochrome P450 (CYP) 1A1, CYP1A2 and NADPH:CYP oxidoreductase (Arlt et al. 2011, 2015a; Stiborova et al. 2001a, 2005, 2012). It has been shown that both AAI and AAII exert their genotoxic and carcinogenic properties by DNA adduct formation and that reductive metabolic activation is a prerequisite for generation of DNA adducts in vivo and in vitro (Gokmen et al. 2013; Schmeiser et al. 2009).

Deduced from the structures of the AA-DNA adducts characterised spectroscopically, we have proposed that a *N*-acylnitrenium ion with a delocalised positive charge is the ultimate carcinogen binding covalently to DNA and forming DNA adducts (Stiborova et al. 2014b). This pentacyclic aristolactam cation ion is formed during partial reduction of the nitro group to the *N*-hydroxy derivative (*N*-hydroxyaristolactam) after condensation with the carboxylic acid moiety in the *peri* position is the direct precursor for binding to the exocyclic amino group of nucleobases via the C7 position (Fig. 1a). The major AA-DNA adducts have been identified as: 7-(deoxyadenosin-*N* ^6^-yl)aristolactam I (dA-AAI); 7-(deoxyguanosin-*N* ^2^-yl)aristolactam I (dG-AAI); 7-(deoxyadenosin-*N* ^6^-yl)aristolactam II (dA-AAII); and 7-(deoxyguanosin-*N* ^2^-yl)aristolactam II (dG-AAII) (Schmeiser et al. 2009, 2014). Characteristic AT to TA transversion mutations have been found in urothelial tumours of AAN patients highlighting the role of dA-AAI adducts as critical premutagenic lesions in AA malignancy (Arlt et al. 2007; Lord et al. 2004; Nik-Zainal et al. 2015; Poon et al. 2013).

The *N*-hydroxyaristolactams I and II have been detected in the urine of AA-treated rats (Chan et al. 2007) indicating that they are rather stable compounds. This was confirmed by the study of Sidorenko et al. (2014) which showed that both *N*-hydroxyaristolactams did not react with DNA in vitro at pH 5.8 in the presence or absence of zinc dust as a reducing agent. It is well known that conjugation reactions catalysed by phase II enzymes are often involved in the activating metabolism of carcinogenic nitroaromatics (Arlt et al. 2005; Glatt and Meinl 2004; Glatt et al. 2016; Rendic and Guengerich 2012). Phase II metabolites of AA have been found in the urine and faeces of AA-treated rodents and include the *N*- and *O*-glucuronide of aristolactam Ia and the *O* ^*8*^-glucuronide, the *O* ^*8*^-acetate and *O* ^*8*^-sulfonated ester of AAIa (Chan et al. 2007); AAIa is considered to be a detoxification product (Levova et al. 2011; Stiborova et al. 2012). As shown for other aromatic hydroxylamines (Glatt and Meinl 2004; Glatt et al. 2016), like those generated during the metabolism of 3-nitrobenzanthrone (3-NBA) (Arlt et al. 2002b, 2003, 2005), the *N*-hydroxyaristolactams may be further activated by *O*-acetylation or *O*-sulfonation to reactive esters capable of generating the same electrophilic species (aristolactam-nitrenium ions) as formed by direct cleavage of the hydroxyl group. However, such aristolactam-*N*-oxy-esters have not yet been identified in studies of the metabolism of AA, which may be owed to their high reactivity.

Some of us recently reported that phase II reactions do not play a role in the bioactivation of AAI; neither native enzymes present in human cytosol nor human recombinant sulfotransferase (e.g. SULT1A enzymes) nor *N*,*O*-acetyltransferases (NATs) enhanced AAI-DNA adduct levels in vitro (Stiborova et al. 2011). In contrast, Meinl et al. (2006) demonstrated that expression of human SULTs (mainly SULT1A1) increased the mutagenic activity of the natural mixture AA in bacterial and mammalian cells. Moreover, Sidorenko et al. (2014) showed that *O*-sulfonated and *O*-acetylated *N*-hydroxyaristolactam I and II readily form DNA adducts in vitro and that binding of *N*-hydroxyaristolactam I and II to DNA was stimulated by mouse cytosol in the presence of 3′-phosphoadenosine-5′-phosphosulfate (PAPS), the cofactor for SULT enzymes. Furthermore, analysis showed that human SULT1B1, SULT1A1 and SULT1A2 can stimulate DNA adduct formation by *N*-hydroxyaristolactam I and II (Sidorenko et al. 2014).

The primary aim of the present study was to evaluate the bioactivation of AAI and AAII mediated by human SULT1A1 in a transgenic mouse model. We employed a transgenic mouse line carrying the functional human *SULT1A1*-*SULT1A2* gene cluster (Dobbernack et al. 2011). In addition, we used *Sult1a1(−/−)* and *Sult1d1(−/−)* mouse lines (Bendadani et al. 2014a; Herrmann et al. 2014). DNA adduct formation in vivo and in vitro was investigated by the ^32^P-postlabelling method.

## Materials and methods

### Carcinogens

Aristolochic acid mixture was purchased from ACROS Organics (#226095000). 3-NBA (CAS number 17117-34-9) was prepared as previously reported (Arlt et al. 2002b).

### Isolation of AAI and AAII

AAI (CAS number 313-67-7) and AAII (CAS number 475-80-9) were purified from the commercially available AA mixture by reverse phase chromatography. Injections (1 mL) of AA mixture, at 10 mg/mL concentration in acetonitrile/25 mM triethylammonium acetate (TEA) mix, were made onto a Gemini column (10 μm, 250 × 21.2 mm, C18, Phenomenex, USA). Chromatographic separation at room temperature was carried out using 322 HPLC pump (Gilson, USA) over a 15-min gradient elution from 25:75 to 40:60 acetonitrile/25 mM TEA at a flow rate of 20 mL/min. Collection was triggered by UV signal (acquired at 254 nm) and collected using a Gilson GX-281 Liquid Handler system (Gilson, USA). The resulting TEA salts of AAI and AAII were converted back to the free acids using HCl. Addition of molar equivalent of NaOH produced the sodium salts which were freeze-dried for use. The purity of AAI and AAII (as sodium salts) was checked using 1D and 2D NMR and high-resolution mass spectrometry (HRMS) which confirmed the compound structures.

### Mouse lines

Wild-type (WT) FVB/N mice (subsequently termed WT mice) were purchased from Harlan (Borchen, Germany). The generation of transgenic FVB/N mice with multiple copies of the human *SULT1A1‒SULT1A2* gene cluster integrated in chromosome 9 has been described elsewhere (Dobbernack et al. 2011). The line termed tg1 in the original study was used. The homozygous transgenic line was bred with WT mice to generate animals with a hemizygous gene status with respect to the human transgene (subsequently termed *hSULT1A1/2*). *Sult1a1(−/−)* and *Sult1d1(−/−)* mice were constructed as described elsewhere (Bendadani et al. 2014a; Herrmann et al. 2014).

### Animal treatment

All animal experiments were conducted in accordance with the law at the German Institute of Human Nutrition (DIfE) Potsdam-Rehbrücke, Nuthetal, Germany, after approval by the Landesamt für Umwelt, Gesundheit und Verbraucherschutz of the State of Brandenburg (reference 23-2347-18-2009). Animals were maintained under timed lighting conditions. Food (Altromin C1000 pellets, Altromin, Germany) and water were available ad libitum. Groups male mice (*n* = 4; 8–10 weeks of age) were used throughout the study. WT, *hSULT1A1/2*, *Sult1a1(−/−)* and *Sult1d1(−/−)* mice were treated with a single dose of 50 mg/kg body weight AAI or AAII by oral gavage according to treatment protocols used previously to study AA metabolism (Arlt et al. 2011; Levova et al. 2011; Stiborova et al. 2012). AAI and AAII (as sodium salts) were dissolved in water at a concentration of 5 mg/mL. Control mice received gavage of solvent (water) only. Similarly, WT and *hSULT1A1/2* mice were injected i.p. with a single dose of 2 mg/kg body weight of 3-NBA according to treatment protocols used previously to study 3-NBA metabolism (Arlt et al. 2005; Krais et al. 2016a; Kucab et al. 2016). 3-NBA was dissolved in tricaprylin at a concentration of 0.2 mg/mL. Control animals received solvent (tricaprylin) only. Animals were killed 24 h post-treatment, and their kidney, bladder, liver, lung, forestomach, glandular stomach, small intestine and colon were removed, snap-frozen in liquid nitrogen and stored at −80 °C until further analysis. DNA was isolated from tissue by a standard phenol–chloroform extraction method.

### Detection of DNA adducts by ^32^P-postlabelling

AAI- and AAII-DNA adduct formation was analysed by the nuclease P1 enrichment version of the ^32^P-postlabelling method as described (Schmeiser et al. 1996, 2012). Resolution of ^32^P-labelled adducts was performed by polyethyleneimine-cellulose (PEI) thin-layer chromatography (TLC) using the following chromatographic conditions: D1: 1 M sodium phosphate, pH 6.5; D3: 3.5 M lithium formate, 8.5 M urea, pH 4.0; D4: 0.8 M lithium chloride, 0.5 M Tris–HCl, 8.5 M urea, pH 9.0; D5: 1.7 M sodium phosphate, pH 6.0. After chromatography, TLC plates were scanned using a Packard Instant Imager (Dowers Grove, IL, USA), and DNA adduct levels (RAL, relative adduct labelling) were calculated as described (Schmeiser et al. 2013). Results were expressed as DNA adducts/10^8^ normal nucleotides. AA-DNA adducts were identified using reference compounds as described (Schmeiser et al. 1996). Urothelial DNA samples from AAN patients were included in the analysis for comparison (Nortier et al. 2000; Schmeiser et al. 2014). For 3-NBA, the butanol-enrichment version of the ^32^P-postlabelling assay was performed to determine DNA adduct formation (Arlt et al. 2002b; Phillips and Arlt 2014). Chromatographic conditions for TLC were: D1: 1.0 M sodium phosphate, pH 6.0; D3: 4.0 M lithium formate, 7.0 M urea, pH 3.5; D4: 0.8 M lithium chloride, 0.5 M Tris, 8.5 M urea, pH 8.0. Using Instant Imager technology, DNA adduct levels (i.e. RAL) were calculated as described (Arlt et al. 2002b) and expressed as DNA adducts/10^8^ nucleotides. 3-NBA-derived DNA adducts were identified as reported (Arlt et al. 2001, 2006).

### Expression of Sult1a1/SULT1A1 and Nqo1 by Western blotting

For the preparation of whole tissue protein, tissues (5–10 mg) of WT and *hSULT1A1/2* mice treated with AAI and AAII were homogenised in 300 µL of T-PER™ buffer supplemented with 1 % Halt™ Protease Inhibitor (both from Thermo Scientific). Samples were sonicated and centrifuged for 20 min at 13,000*g* at 4 °C. The supernatant was removed and protein concentration was determined using the Pierce^®^ BCA protein assay kit (Thermo Scientific, USA) according to manufacturer instructions. Protein samples (20 µg) were denatured with ß-mercaptoethanol at 80 °C for 10 min prior to loading onto 10 % Bis–Tris 26-well NuPAGE midi gels. Proteins were separated by sodium dodecyl sulfate–polyacrylamide electrophoresis (SDS-PAGE) in MOPS buffer at 130 V and subsequently transferred onto a nitrocellulose membrane at 100 V for 2 h. The membrane was blocked in 3 % w/v non-fat milk dissolved in 0.2 % TBS-T (Tris-buffered saline containing 0.2 % Tween-20), then probed overnight at 4 °C with the following antibodies: Human SULT1A1 was detected with antisera raised in rabbits against bacterial inclusion bodies of human SULT1A 1:10,000; (Martin et al. 2010), mouse Nqo1 was detected with rabbit pAb #N5288 (1:5000; Sigma). Peroxidase-conjugated goat anti-rabbit (#170-5046; 1:10,000; Bio-Rad) was used as secondary antibody. Gapdh was detected with mouse mAb #MAB374 (1:10,000; Millipore) in 3 % milk at room temperature for 1 h and peroxidase-conjugated goat anti-mouse as secondary anti-mouse (#170-5047; 1:10,000; Bio-Rad). All proteins were visualised using enhanced chemiluminescence SuperSignal West Pico detection reagent (#34080; Thermo Scientific).

### Preparation of cytosols

Hepatic and renal cytosols were isolated as described (Arlt et al. 2008, 2015b; Krais et al. 2016a, b). Because treating the mice with AAI or AAII might influence levels and activities of xenobiotic-metabolising enzymes (XMEs), cytosols were isolated from organs of both control (vehicle-treated) and AAI-AAII-treated mice (see above). Pooled cytosolic fractions (*n* = 4 mice per group) were used for further analysis. Small aliquots were stored at −80 °C until use.

### Cytosolic incubations used for AAI-DNA adduct analysis

The de-aerated and nitrogen-purged incubation mixtures, in which cytosols were used to activate AAI, contained 50 mM Tris–HCl buffer (pH 7.4), 0.2 % Tween 20, cofactors for cytosolic enzymes Nqo1 and sulfotransferase (1 mM NADPH with or without 100 µM PAPS), 1 mg mouse hepatic or renal cytosolic protein, 0.5 mg calf thymus DNA (2 mM dNp) and 0.5 mM AAI in a final volume of 750 µL. Incubations with cytosols were performed at 37 °C for 60 min; AAI-derived DNA adduct formation was found to be linear up to 2 h (Stiborova et al. 2003). Control incubations were performed either (1) without cytosol, (2) without cofactors (NADPH, PAPS), (3) without DNA or (4) without AAI. After extraction with ethyl acetate DNA was isolated from the residual water phase by a standard phenol/chloroform extraction method. AAI-DNA adduct formation was analysed by ^32^P-postlabelling as described above.

### Determination of Nqo1 activity in cytosolic fractions

Nqo1 activity was measured in hepatic and renal cytosols using menadione (2-methyl-1,4-naphthoquinone) as a substrate as described previously (Levova et al. 2011, 2012; Mizerovska et al. 2011). The assay was improved by the addition of cytochrome *c* and Nqo1 activity expressed as nmol cytochrome *c* reduced.

### Determination of sulfotransferase activity in cytosolic fractions

Sulfotransferase enzyme activity was characterised in renal and hepatic cytosolic samples by monitoring the formation of *p*-nitrophenol from a 5′-phosphoadenosine 3′-phosphosulfate (PAPS)-regenerating system (Frame et al. 2000; Krais et al. 2016b). It is based on the catalysed synthesis of 2-naphthylsulphate from 2-naphthol and PAPS through sulfotransferase while PAPS is continuously regenerated by using *p*-nitrophenyl sulphate as sulfo-group donor. In a 96-well plate the incubation mixture (200 µL per well) contained 48 mM sodium phosphate buffer (pH 7.4), 20 µM PAPS, 5 mM *p*-nitrophenyl sulfate potassium salt, 0.1 mM 2-naphthol, 1 mM MgCl_2_ and 400–600 µg protein of cytosolic fraction. Shortly before the measurement, the reaction was initiated by the addition of 2-naphthol. The colorimetric formation of *p*-nitrophenol was detected by its absorbance at 405 nm in kinetic manner every 2 min for 60 min on a Synergy HT Plate Reader (Bio-TEK Instruments). Enzyme activities were calculated as nmol *p*-nitrophenol per min/mg protein.

### Cytosolic incubations used for 3-NBA-derived DNA adduct analysis

The de-aerated and nitrogen-purged incubation mixtures, in a final volume of 750 µL, consisted of 50 mM Tris–HCl buffer (pH 7.4), containing 0.2 % Tween 20, cofactors for cytosolic enzymes Nqo1 and Sult/SULT (1 mM NADPH with or without 100 µM PAPS), 1 mg of mouse hepatic cytosolic protein, 100 µM 3-NBA (dissolved in 7.5 µL dimethylsulfoxide [DMSO]) and 0.5 mg of calf thymus DNA. The reaction was initiated by adding 3-NBA. Incubations with human cytosols were carried out at 37 °C for 3 h; the cytosol-mediated 3-NBA-derived DNA adduct formation was found to be linear up to 4 h (Arlt et al. 2005). Control incubations were carried out (1) without activating system (cytosol), (2) without cofactors (NADPH, PAPS), (3) without DNA or (4) without 3-NBA. After extraction with ethyl acetate, DNA was isolated from the residual water phase by phenol/chloroform extraction. 3-NBA-derived DNA adduct formation was analysed by ^32^P-postlabelling as described above.

### Statistical analysis

Statistical analyses were performed with Prism GraphPad Software (version 6.04), and *P* < 0.05 was considered significant.

## Results

### DNA adduct formation in WT versus *hSULT1A1/2* mice treated with AAI, AAII and 3-NBA

**Fig. 2:**
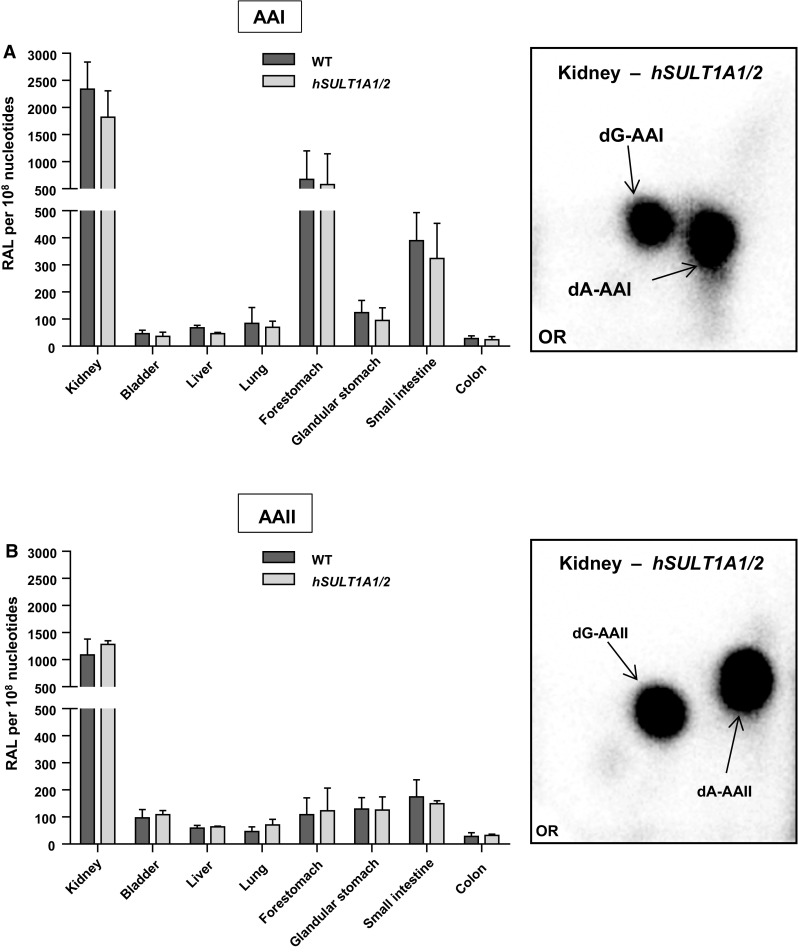
Total DNA adduct levels measured by the nuclease P1 enrichment version of the ^32^P-postlabelling method in various organs of WT and *hSULT1A1/2* mice after exposure to a single oral dose of 50 mg/kg body weight AAI (**a**) or AAII (**b**). Values are the mean ± SD (*n* = 4 animals). Statistical analysis was performed by Student’s *t* test; no significant differences were observed between WT and *hSULT1A1/2* mice. *Inserts* Autoradiograms of DNA adducts, measured by ^32^P-postlabelling, in kidney tissue of *hSULT1A1/2* mice. These profiles are representative of adduct pattern obtained with DNA from other mouse tissues including bladder, liver, lung, forestomach, glandular stomach, small intestine and colon, and those in WT mice. The origin (OR) on the TLC plate, at the bottom left-hand corners, was cut off before exposure. 7-(deoxyadenosin-*N* ^6^-yl)-aristolactam I (dA-AAI); 7-(deoxyguanosin-*N* ^2^-yl)-aristolactam I (dG-AAI); 7-(deoxyadenosin-*N* ^6^-yl)-aristolactam II (dA-AAII); 7-(deoxyguanosin-*N* ^2^-yl)-aristolactam II (dG-AAII)

AAI-/AAII-DNA adduct formation was determined by ^32^P-postlabelling in kidney, bladder, liver, lung, forestomach, glandular stomach, small intestine and colon of WT and *hSULT1A1/2* mice treated with a single oral dose of 50 mg/kg body weight AAI or AAII for 24 h. The adduct pattern induced by AAI and AAII were qualitatively similar in all organs of both mouse lines tested. The pattern induced by AAI consisted of two major adduct spots, identified previously (Arlt et al. 2002c) as 7-(deoxyadenosin-*N* ^6^-yl)-aristolactam I (dA-AAI) and 7-(deoxyguanosin-*N* ^2^-yl)-aristolactam I (dG-AAI) (see insert Fig. 2a). Similarly, after treatment with AAII two observed major adduct spots were identified (Arlt et al. 2002c) as 7-(deoxyadenosin-*N* ^6^-yl)-aristolactam II (dA-AAII) and 7-(deoxyguanosin-*N* ^2^-yl)-aristolactam II (dG-AAII) (see insert Fig. 2b). dA-AAI, dG-AAI and dA-AAII have been found in urothelial tissue of AAN patients (Arlt et al. 2002a; Bieler et al. 1997; Nortier et al. 2000; Schmeiser et al. 2012). No DNA adducts were detected in control (vehicle-treated) animals (data not shown).

Total AAI-DNA adduct levels found in organs ranged from 25 to 2300 adducts per 10^8^ nucleotides (Fig. 2a). Highest DNA binding was observed in the kidney where adduct levels were ~35-fold higher than in liver, followed by forestomach and small intestine, but there were no significant differences between both mouse lines in DNA adduct formation in any of the tissues investigated (Fig. 2a). Similarly no differences in DNA binding between mouse lines were observed after AAII treatment (Fig. 2b). Again, total AAII-DNA adduct formation was the highest in the kidney (~1300 adducts per 10^8^ nucleotides) with adduct levels being much lower in all other tissues. It is noteworthy that AAI-DNA adduct levels in forestomach and small intestine were much higher compared to levels of DNA adduct induced by AAII in these tissues. Collectively, these findings indicate that expression of human SULT1A does not contribute to the bioactivation of AAI and AAII in vivo.

**Fig. 3:**
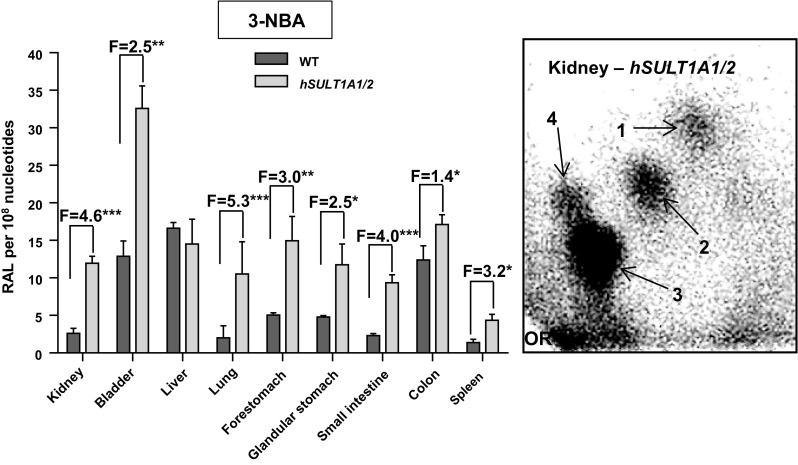
Total DNA adduct levels measured in various organs of WT and *hSULT1A1/2* mice after exposure to a single i.p. dose of 2 mg/kg body weight 3-NBA. DNA adduct formation was determined by the butanol-enrichment version of the ^32^P-postlabelling method. Values are the mean ± SD (*n* = 3 animals). *F*, fold difference in DNA binding relative to WT mice. Statistical analysis was performed by Student’s *t* test (*P < 0.05, **P < 0.01, ***P < 0.001; different from WT mice). *Insert* Autoradiogram of DNA adducts, measured by ^32^P-postlabelling, in kidney tissue of *hSULT1A1/2* mice. These profiles are representative of adduct pattern obtained with DNA from other mouse tissues including bladder, liver, lung, forestomach, glandular stomach, small intestine and colon, and those in WT mice. The origin (OR) on the TLC plate, at the bottom left-hand corners, was cut off before exposure. Spot 1, 2-(2′-deoxyadenosin-*N* ^6^-yl)-3-aminobenzanthrone (dA-*N* ^6^-3-ABA); spot 2, as-yet-uncharacterised deoxyadenosine adduct; spot 3, 2-(2′-deoxyguanosin-*N* ^2^-yl)-3-aminobenzanthrone (dG-*N* ^2^-3-ABA); and spot 4, *N*-(2′-deoxyguanosin-8-yl)-3-aminobenzanthrone (dG-C8-*N*-3-ABA)

As 3-NBA is activated by SULTs (Arlt et al. 2002b, 2003, 2005), 3-NBA-DNA adduct formation in the same mouse lines was measured by ^32^P-postlabelling in kidney, bladder, liver, lung, forestomach, glandular stomach, small intestine and colon after 3-NBA treatment with a single i.p. dose of 2 mg/kg body weight for 24 h. We have shown previously that metabolic activation of 3-NBA occurs after initial nitroreduction catalysed by cytosolic nitroreductases (e.g. NQO1) leading to *N*-hydroxy-3-aminobenzanthrone (*N*–OH-3-ABA) (Fig. 1b) (Arlt 2005). The genotoxicity (i.e. DNA adduct formation) of *N*–OH-3-ABA is enhanced by the expression of SULT1A1 or SULT1A2 in bacterial and mammalian cells in culture (Arlt et al. 2002b, 2003, 2005). Previous studies have also shown that enrichment by butanol extraction yields more adduct spots and a better recovery of 3-NBA-DNA adducts than using enrichment by nuclease P1 digestion (Arlt et al. 2001). The adduct pattern induced by 3-NBA consisted of a cluster of four major adducts (spots 1‒4) in all tissues tested (see insert Fig. 3). These were characterised previously as 2-(2′-deoxyadenosin-*N* ^6^-yl)-3-aminobenzanthrone (dA-*N* ^6^-3-ABA; spot 1), 2-(2′-deoxyguanosin-*N* ^2^-yl)-3-aminobenzanthrone (dG-*N* ^2^-3-ABA; spot 3) and *N*-(2′-deoxyguanosin-8-yl)-3-aminobenzanthrone (dG-C8-*N*-3-ABA); spot 4), while spot 2 is an as-yet-uncharacterised deoxyadenosine adduct (Arlt et al. 2001, 2006). No DNA adducts were observed in DNA isolated from tissue of control animals treated with vehicle only (tricaprylin) (data not shown).

Except for the liver, compared to WT mice DNA binding by 3-NBA was up to ~fivefold higher in kidney, bladder, lung, forestomach, glandular stomach, small intestine and colon of *hSULT1A1/2* mice (Fig. 3). These findings demonstrate that the expression of human SULT1A increases the activation of 3-NBA in several organs in vivo.

### Organ-specific expression of human SULT1A1/2 and mouse Nqo1 proteins in WT and *hSULT1A1/2* mice treated with AAI and AAII

**Fig. 4:**
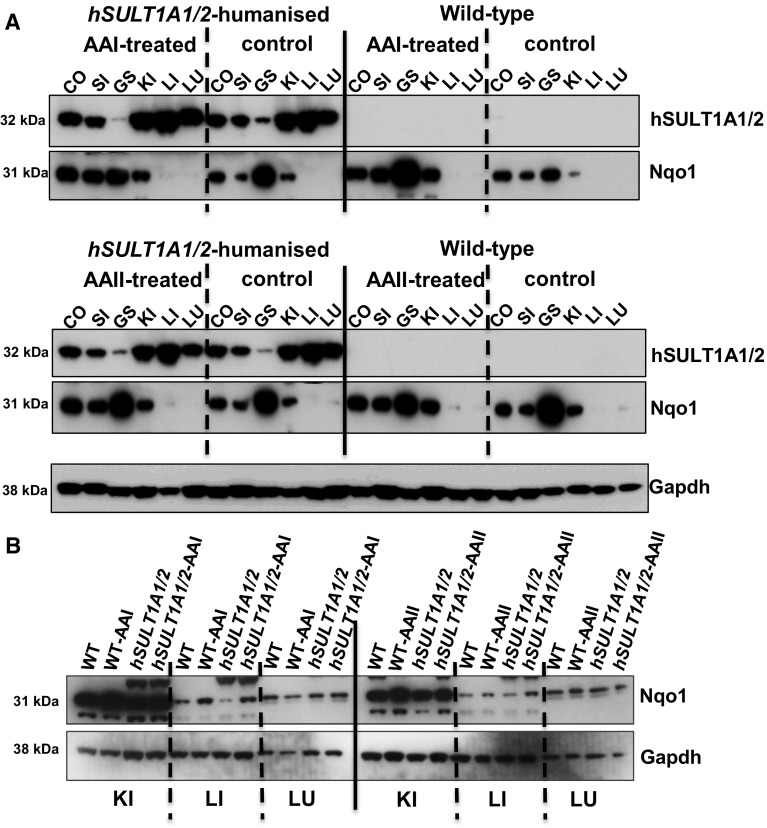
**a** Western blot analysis of human SULT1A1/2 and murine Nqo1 protein expression in whole tissue lysates isolated from WT and *hSULT1A1/2* mice after exposure to a single oral dose of 50 mg/kg body weight AAI or AAII. **b** Western blot analysis of murine Nqo1 protein expression in whole tissue lysates isolated from the kidney, liver and lung of WT and *hSULT1A1/2* mice after exposure to AAI or AAII. Gapdh was used as loading control and representative blots are shown. *CO* colon, *SI* small intestine, *GS* glandular stomach, *KI* kidney, *LI* liver, *LU* lung, *SPL* spleen. Representative images of the Western blotting are shown, and at least duplicate analysis was performed from independent experiments

In order to verify the expression of human SULT1A1/2 proteins in the *hSULT1A1/2* mice treated with AAI and AAII we used Western blotting (Fig. 4). Immunoblotting using anti-hSULT1A antisera that cross-reacted only marginally with murine Sult1a proteins was performed in colon, small intestine, glandular stomach, kidney, liver and lung. No tissue for analysis was left from forestomach and bladder as the whole organs were used for DNA adduct analysis. The tissue-specific expression of human SULT1A1/2 proteins in *hSULT1A1/2* mice was qualitatively similar after treatment with AAI and AAII (Fig. 4a). No expression of human SULT1A1/2 protein (or a protein that could be confounded with human SULT1A1/2) was detected in WT mice under the experimental conditions used. No induction of human SULT1A1/2 proteins was found after AAI or AAII treatment relative to control (vehicle-treated) mice. Relatively high expression of hSULT1A1/2 proteins was observed in kidney, liver and lung; expression was lower in colon, small intestine and glandular stomach.

Strong expression of murine Nqo1 protein was observed in colon, small intestine, glandular stomach and kidney (Fig. 4a). Interestingly, the strongest expression of Nqo1 was observed in glandular stomach, even though it had the weakest expression of human SULT1A1/2. Overall, the tissue distribution of the Nqo1 protein expression was similar in WT and *hSULT1A1/2* mice, except for glandular stomach where expression levels were more variable (Fig. 4a). In comparison Nqo1 protein levels were relatively low in liver and lung and hardly visible on the blots comparing all tissues (Fig. 4a). Therefore, Nqo1 expression levels in kidney, liver and lung were reanalysed in separate blots using a longer film exposure time. As shown in Fig. 4b, Nqo1 expression was now clearly detectable in those tissues. Liver and kidney Nqo1 was induced by AAI and AAII in both WT and *hSULT1A1/2* mice, whereas this effect was not seen in lung (Fig. 4b).

### DNA adduct formation by AAI in vitro and enzyme activity in renal and hepatic cytosolic fractions isolated from WT and *hSULT1A1/2* mice treated with AAI and AAII

**Fig. 5:**
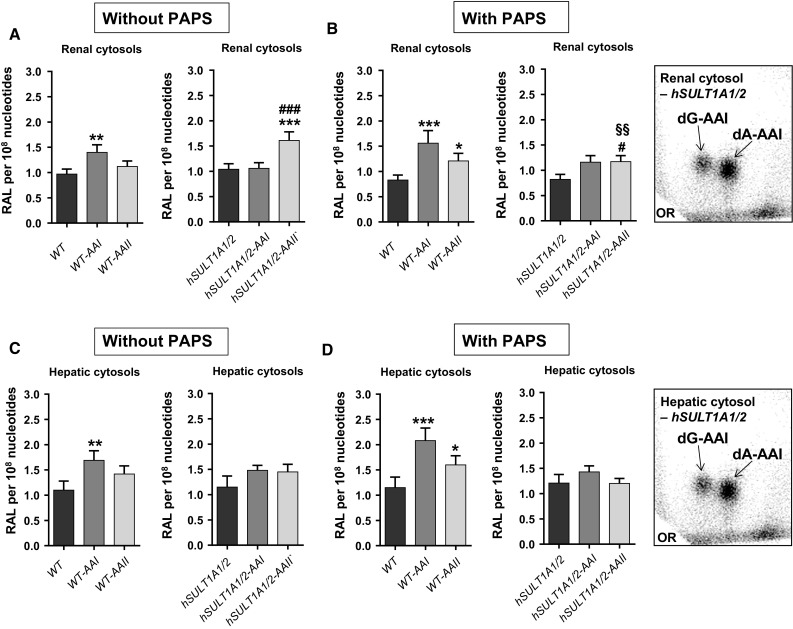
Total AAI-DNA adducts, as measured by the nuclease P1-enrichment version of the ^32^P-postlabelling method, formed ex vivo in renal (**a**, **b**) and hepatic cytosols (**c**, **d**) isolated from untreated, AAI- or AAII-pretreated WT and *hSULT1A1/2* mice incubated with AAI and DNA in the absence (**a** and **c**) or presence of PAPS (**b**, **d**). Values are the mean ± range (*n* = 4; duplicate incubations and each sample was determined by two independent ^32^P-postlabelling analyses). Statistical analysis was performed by two-way ANOVA followed by Tukey’s multiple comparison test (*P < 0.05, **P < 0.01, ***P < 0.001—different from control WT mice; ^#^ *P* < 0.05, ^###^ *P* < 0.001—different from control *hSULT1A1/2* mice; ^§§^ *P* < 0.01—different from incubation without PAPS). *Inserts* Representative autoradiograms of DNA adducts, measured by ^32^P-postlabelling, in AAI incubations with renal and hepatic cytosols. The origin (OR) on the TLC plate, at the bottom left-hand corners, was cut off before exposure. 7-(deoxyadenosine-*N* ^6^-yl)-aristolactam I (dA-AAI); 7-(deoxyguanosin-*N* ^2^-yl)-aristolactam I (dG-AAI); 7-(deoxyadenosine-*N* ^6^-yl)-aristolactam II (dA-AAII); 7-(deoxyguanosin-*N* ^2^-yl)-aristolactam II (dG-AAII)

**Fig. 6:**
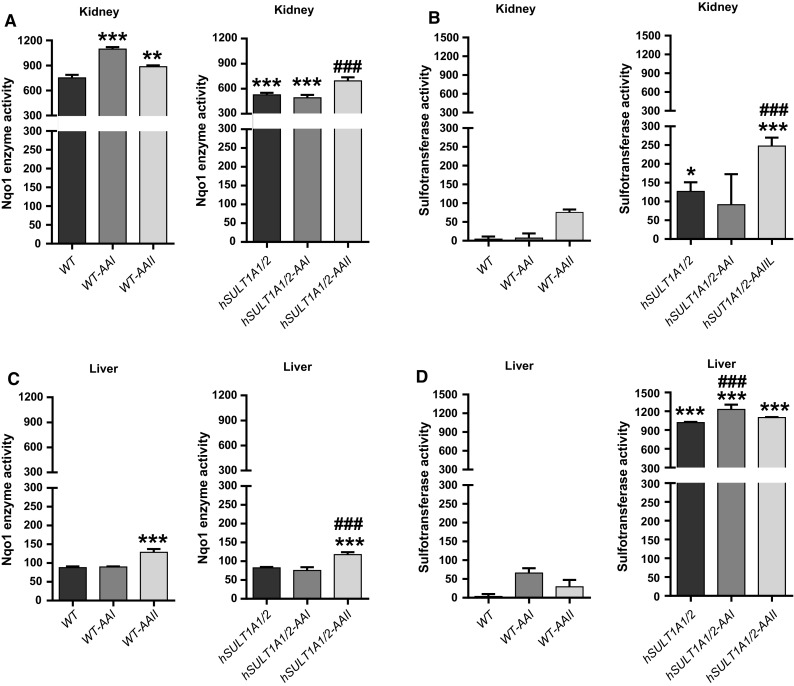
Measurement of Nqo1 (**a**, **c**) and sulfotransferase (**b**, **d**) enzyme activity in cytosolic fractions of the kidneys (*upper panels*) and livers (*lower panels*) from untreated, AAI- or AAII-treated WT and *hSULT1A1/2* mice. Nqo1 enzyme activity was determined using menadione and cytochrome *c* as substrate and expressed as nmol cytochrome *c*/min/mg protein. Sulfotransferase enzyme activity was determined using a colorimetric assay with *p*-nitrophenol sulfate as sulfo-donor and is expressed as nmol *p*-nitrophenol/min/mg protein. Values are the mean ± SD of three determinations. Statistical analysis was performed by two-way ANOVA followed by Tukey’s multiple comparison test (*P < 0.05, **P < 0.01, ***P < 0.001—different from control WT mice; ^###^ *P* < 0.001—different from control *hSULT1A1/2* mice)

In a second set of experiments to investigate the potential role of human SULT1A1/2 on the metabolic activation of AAI, we incubated renal or hepatic cytosolic fractions from WT and *hSULT1A1/2* mice in vitro with DNA and AAI to determine AAI-DNA adduct formation by ^32^P-postlabelling (Fig. 5). Cytosolic incubations were conducted in the presence and absence of PAPS but all contained NADPH as a cofactor. In the absence of PAPS, NADPH-dependent DNA adduct formation of AAI in cytosols was used as a measure of AAI bioactivation by murine Nqo1. Subsequently in the presence of PAPS, any potential alterations in AAI-DNA adduct levels should be attributable to AAI bioactivation by murine Sult1a1/2 in WT mice or human SULT1A1/2 in *hSULT1A1/2* mice. Renal (Fig. 5; *upper panels*) and hepatic (Fig. 5; *lower panels*) cytosols were capable of bioactivating AAI to form DNA adducts, generating the same pattern of AAI-DNA adducts as found in vivo (Fig. 5, inserts). In both renal and hepatic cytosolic fractions of untreated WT and *hSULT1A1/2* mice no difference in AAI-DNA adduct formation was observed after the addition of PAPS (Fig. 5), indicating that neither murine nor human SULTs contribute to AAI bioactivation. This conclusion is in line with the observation that Nqo1 enzyme activity in renal and hepatic cytosolic fractions was similar for both genotypes (Fig. 6a, c), suggesting that AAI-DNA adduct formation in vitro is predominantly catalysed by Nqo1. In contrast, the sulfotransferase activities in renal and hepatic cytosols, measured colorimetrically as sulfo-transfer from *p*-nitrophenol sulfate to 2-naphthol, were manifold higher in *hSULT1A1/2* mice compared to WT mice (Fig. 6b, d). As no difference in AAI-DNA adduct formation was observed in the presence of PAPS, despite massive differences in sulfotransferase activities in cytosolic fractions, these results confirm the conclusion that SULTs do not contribute to the metabolic activation of AAI (i.e. AAI-DNA adduct formation; Fig. 5) under these experimental conditions.

Treatment of mice with AAI and AAII led to an induction of Nqo1 activity in both kidney and liver (Fig. 6a, c). For example, in WT kidney induction of Nqo1 activity was found both after AAI and AAII treatment whereas in liver only AAII was able to induce Nqo1 activity. Induction of sulfotransferase activity was also observed in both kidney and liver after AAI and AAII treatment; the pattern of induction was dependent on the mouse line (i.e. WT or *hSULT1A1/2* mice) and compound used (i.e. AAI or AAII) (Fig. 6b, d). In incubations using cytosolic fractions isolated from AAI- or AAII-treated mice AAI-DNA adduct formation was significantly increased under certain experimental conditions relative to cytosolic incubations from untreated animals (Fig. 5). However, it is noteworthy that the increases in AAI-DNA adduct levels were often relatively small. Under certain experimental conditions AAI-DNA adduct levels observed in vitro seem to correlate with Nqo1 activity (compare Fig. 6a, c). Whereas in renal cytosols isolated from AAI- and AAII-treated mice increased AAI-DNA adduct levels seem to be associated with enhanced Nqo1 activity, these associations were less clear in hepatic cytosols isolated from AAI- and AAII-treated mice. Although both AAI and AAII were capable of inducing sulfotransferase activity in treated animals (Fig. 6b, d), no clear associations can be made to the observed AAI-DNA adduct levels in vitro in cytosolic incubations (compare Fig. 5). For example, given the strong differences in sulfotransferase activities in hepatic cytosols between WT and *hSULT1A1/2* mice in both control (vehicle-treated) and AAI-/AAII-treated animals, these differences are not observed in AAI-DNA adduct formation in vitro in the same cytosolic sample. Thus, it seems unlikely that sulfotransferase detected in the transfer assay make any major contribution to AAI bioactivation leading to DNA adduct formation in vitro.

### DNA adduct formation in WT *versus Sult1a1(−/−)* or *Sult1d1(−/−)* mice treated with AAI and AAII

**Fig. 7:**
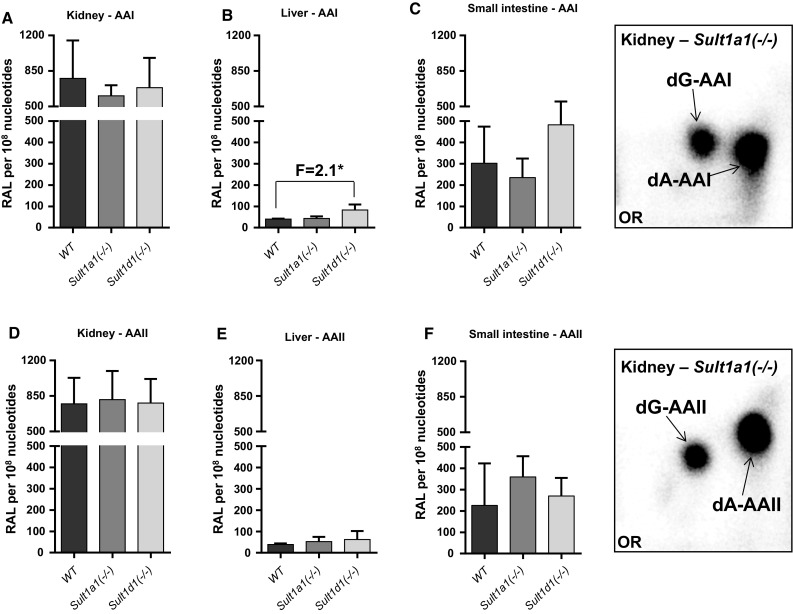
Total DNA adduct levels measured by the nuclease P1 enrichment version of the ^32^P-postlabelling method in kidney (**a**, **d**), liver (**b**, **e**) and small intestine (**c**, **f**) of WT, *Sult1a1(−/−)* and *Sult1d1(−/−)* mice after exposure to a single oral dose of 50 mg/kg body weight AAI (**a**–**c**) or AAII (**d**–**f**). Values are the mean ± SD (*n* = 4 animals). *F*, fold difference in DNA binding relative to WT mice. Statistical analysis was performed by one-way ANOVA followed by Tukey’s multiple comparison test (*P < 0.05—different from WT mice). *Inserts* Autoradiograms of DNA adducts, measured by ^32^P-postlabelling, in kidney tissue of *Sult1a1(−/−)* mice. These profiles are representative of adduct pattern obtained with DNA from liver and small intestine, and those in WT and *Sult1d1(−/−)* mice. The origin (OR) on the TLC plate, at the bottom left-hand corners, was cut off before exposure. 7-(deoxyadenosin-*N* ^6^-yl)-aristolactam I (dA-AAI); 7-(deoxyguanosin-*N* ^2^-yl)-aristolactam I (dG-AAI); 7-(deoxyadenosin-*N* ^6^-yl)-aristolactam II (dA-AAII); 7-(deoxyguanosin-*N* ^2^-yl)-aristolactam II (dG-AAII)

We next studied the role of murine Sults on metabolic activation of AAI and AAII in vivo. AAI-/AAII-DNA adduct formation was determined by ^32^P-postlabelling in kidney, liver and small intestine of WT, *Sult1a1(−/−)* and *Sult1d1(−/−)* mice treated with a single oral dose of 50 mg/kg body weight AAI or AAII for 24 h. Besides Sult1a1 we also studied the potential influence of Sult1d1 as this isoenzyme is also highly expressed in kidney (Alnouti and Klaassen 2006), the target organ of AA (geno)toxicity. The DNA adduct pattern induced by AAI and AAII in WT, *Sult1a1(−/−)* and *Sult1d1(−/−)* mice was the same as that found in *hSULT1A1/2* mice (Fig. 7, inserts).

Total DNA adduct levels after AAI and AAII treatment ranged from 25 to 800 adducts per 10^8^ nucleotides (Fig. 7). Highest DNA binding was observed in the kidney after both AAI and AAII exposure. It should be noted that the treatment of *hSULT1A1/2* (compare Fig. 2) and knockout animals (Fig. 7) with AAI and AAII was done on separate occasions and that DNA adduct analyses for both treatment sets were also done separately. These are possible reasons to explain the differences in DNA adduct levels observed in WT animals between sample sets; in AAI-treated kidney samples the difference is more pronounced than in AAII-treated kidney samples. In kidney knockout of Sult1a1 or Sult1d1 had no impact on AAI- or AAII-DNA adduct formation (Fig. 7a, d). Similarly, no differences in DNA adduct formation between mouse lines were found in liver (Fig. 7b, e) and small intestine (Fig. 7c, f), except for AAI-treated *Sult1d1(−/−)* mice where DNA binding was ~twofold higher in liver relative to WT. Collectively, these results demonstrate that in kidney murine Sults (i.e. Sult1a1 or Sult1d1) do not contribute to AA genotoxicity (i.e. DNA adduct formation) in vivo.

### DNA adduct formation by AAI and 3-NBA in vitro and enzyme activity in renal and hepatic cytosolic fractions isolated from WT, *Sult1a1(−/−)* or *Sult1d1(−/−)* mice treated with AAI and AAII

**Fig. 8:**
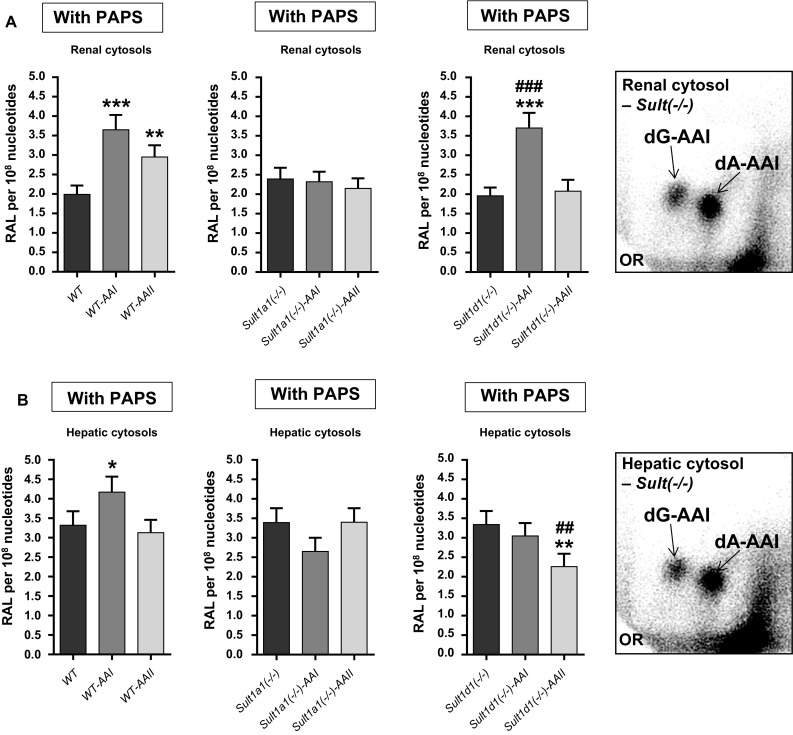
Total AAI-DNA adduct levels, as measured by the nuclease P1-enrichment version of the ^32^P-postlabelling method, formed ex vivo in renal (**a**) and hepatic cytosols (**b**) isolated from untreated, AAI- or AAII-pretreated WT, *Sult1a1(−/−)* and *Sult1d1(−/−)* mice incubated with AAI and DNA in the presence of PAPS. Values are the mean ± range (*n* = 4 analyses; duplicate incubations and each sample was determined by two independent ^32^P-postlabelling analyses). Statistical analysis was performed by two-way ANOVA followed by Tukey’s multiple comparison test (*P < 0.05, **P < 0.01, ***P < 0.001—different from control WT mice; ^##^ *P* < 0.01, ^###^ *P* < 0.001—different from control *Sult1d1(−/−)* mice). *Inserts* Representative autoradiograms of DNA adducts, measured by ^32^P-postlabelling, in AAI incubations with renal and hepatic cytosols. The origin (OR) on the TLC plate, at the bottom left-hand corners, was cut off before exposure. 7-(deoxyadenosine-*N* ^6^-yl-aristolactam I (dA-AAI); 7-(deoxyguanosin-*N* ^2^-yl-aristolactam I (dG-AAI); 7-(deoxyadenosine-*N* ^6^-yl-aristolactam II (dA-AAII); 7-(deoxyguanosin-*N* ^2^-yl-aristolactam II (dG-AAII)

**Fig. 9:**
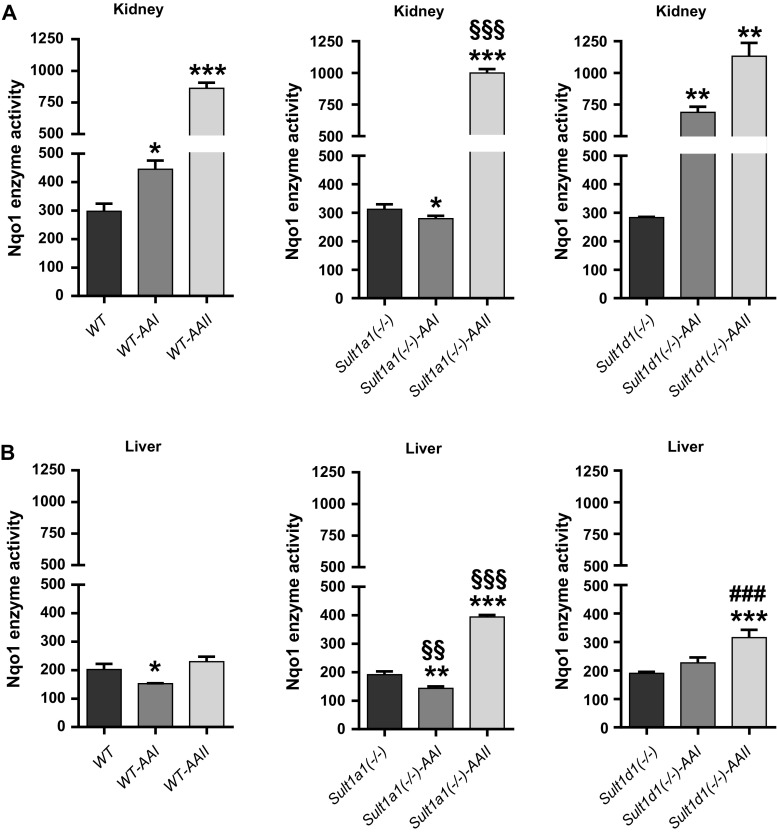
Nqo1 enzyme activity in cytosolic fractions of the kidneys (**a**) and livers (**b**) from untreated, AAI- or AAII-treated WT, *Sult1a1(−/−)* and *Sult1d1(−/−)* using menadione and cytochrome *c* as substrate. Values are the mean ± SD of three determinations; Nqo1 enzyme activity is expressed as nmol cytochrome *c*/min/mg protein. Statistical analysis was performed by two-way ANOVA followed by Tukey’s multiple comparison test (*P < 0.05, **P < 0.01, ***P < 0.001—different from control WT mice; ^§§^ *P* < 0.01, ^§§§^ *P* < 0.001—different from control *Sult1a1(−/−)* mice; ^##^ *P* < 0.01, ^###^ *P* < 0.001—different from control *Sult1d1(−/−)* mice, respectively)

**Fig. 10:**
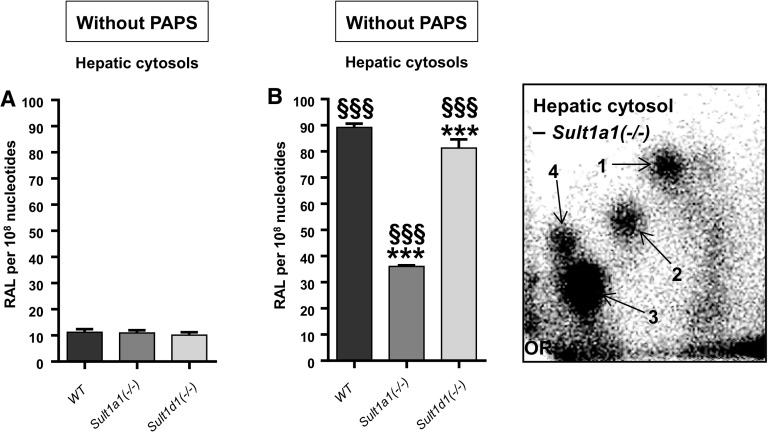
Total 3-NBA-DNA adduct levels, as measured by the butanol-enrichment version of the ^32^P-postlabelling method, formed in vitro in hepatic cytosols isolated from WT, *Sult1a1(−/−)* and *Sult1d1(−/−)* mice incubated with 3-NBA and DNA in the absence (**a**) or presence of PAPS (**b**). Values are the mean ± range (*n* = 4 analyses; duplicate incubations and each sample was determined by two independent ^32^P-postlabelling analyses). Statistical analysis was performed by two-way ANOVA followed by Tukey’s multiple comparison test (***P < 0.001—different from WT mice; ^§§§^ *P* < 0.001—different from incubation without PAPS). *Insert* Representative autoradiogram of DNA adducts, measured by ^32^P-postlabelling, in AAI incubations with hepatic cytosols. The origin (OR) on the TLC plate, at the bottom left-hand corners, was cut off before exposure. Spot 1, 2-(2′-deoxyadenosin-*N* ^6^-yl)-3-aminobenzanthrone (dA-*N* ^6^-3-ABA); spot 2, as-yet uncharacterised deoxyadenosine adduct; spot 3, 2-(2′-deoxyguanosin-*N* ^2^-yl)-3-aminobenzanthrone (dG-*N* ^2^-3-ABA); and spot 4, *N*-(2′-deoxyguanosin-8-yl)-3-aminobenzanthrone (dG-C8-*N*-3-ABA)

Hepatic and renal cytosols isolated from AAI-/AAII-pretreated mice of all lines were incubated with AAI in the presence of DNA and the enzymatic cofactors NADPH and PAPS. We anticipated that any involvement of Sults in AAI bioactivation would result in diminished AAI-DNA adduct formation in incubations using cytosolic fractions from *Sult*-knockout mouse lines. Renal (Fig. 8a) and hepatic (Fig. 8b) cytosols were capable of bioactivating AAI to form DNA adducts, generating the same pattern of AAI-DNA adducts formed in vivo (Fig. 8, inserts). With both renal and hepatic cytosolic fractions of untreated WT, *Sult1a1(−/−)* and *Sult1d1(−/−)* mice no difference in AAI-DNA adduct formation was observed in the presence of PAPS (Fig. 8), indicating that neither Sult1a1 nor Sult1d1 contributes to AAI bioactivation. This conclusion was in line with the observation that Nqo1 activity in renal and hepatic cytosolic fractions was similar for all three genotypes (Fig. 9), suggesting that AAI-DNA adduct formation in vitro is predominantly catalysed by Nqo1. In contrast, when hepatic cytosols isolated from the same mouse lines were incubated with 3-NBA in the presence of DNA, NADPH and PAPS, 3-NBA-induced DNA adduct levels were diminished by ~60 % in *Sult1a1(−/−)* cytosolic incubations relative to WT (Fig. 10b). These findings confirmed previous in vitro studies on the important role of SULT1A1 in 3-NBA genotoxicity (Arlt et al. 2001, 2003, 2005). The contribution of murine Sults in the bioactivation of 3-NBA was also demonstrated by the fact that 3-NBA-DNA adduct formation was up to ~eightfold higher in incubations in the presence of PAPS compared to incubations without PAPS (Fig. 10). The pattern of 3-NBA-DNA adducts generated in vitro was the same as that found in vivo (Fig. 10, insert). As shown in Fig. 10b disruption of Sult1d1 had no impact on 3-NBA-DNA adduct formation relative to WT.

The results obtained in incubations using cytosolic fractions isolated from AAI- or AAII-pretreated mice were more complex. As shown in Fig. 9 treatment of mice with AAI and AAII led to the induction of Nqo1 activity (compare also Fig. 6a, c). However, increased Nqo1 activity did not always correlate with the AAI-DNA adduct levels observed in in vitro incubations (compare Figs. 8 and 9). In other cytosolic incubations such as *Sult1a1(−/−)*-*AAI* and *Sult1d1(−/−)*-*AAII* (Fig. 9b) AAI-DNA adduct formation was actually lower compared to cytosolic incubations from control (vehicle-treated) mice of the same genotype. However, observed changes were relatively small, so it is difficult to draw any solid conclusions from these observations.

## Discussion

A powerful tool of elucidating the activation pathway of chemical carcinogens is to analyse their ability to produce covalent DNA adducts and to determine what factors either enhance or inhibit adduct formation (Phillips 2013). This approach has been successfully used by Stiborova and co-workers to assess the bioactivation of AA (Arlt et al. 2015a; Stiborova et al. 2013, 2014a) and was therefore also employed in the present study. For the detection of DNA adducts we used the ^32^P-postlabelling method because the chemical structures of AA-DNA adducts observed in this assay have been identified. As for other nitroaromatics (Arlt 2005), nitroreduction is the major activation pathway of AAI and AAII that is catalysed by a number of cytosolic and microsomal enzymes, cytosolic NQO1 being the most efficient nitroreductase (Stiborova et al. 2001b, 2002, 2003). Following activation by NQO1 both AAI and AAII produce specific DNA adducts identical to those found in humans (Nortier et al. 2000; Schmeiser et al. 1996, 2012, 2014), confirming that nitroreduction is the crucial step in the bioactivation of AA (Martinek et al. 2011; Stiborova et al. 2011).

Recently we reported that, in contrast to NQO1, human cytosolic phase II enzymes such as *N*,*O*-acetyltransferases (NATs) and SULTs do not play a role in AAI activation in cell-free in vitro systems (Stiborova et al. 2011). Neither native enzymes present in human cytosol nor human recombinant sulfotransferase enhanced AAI-DNA adduct levels in vitro. Likewise, cofactors of NAT and SULT enzymes, acetyl-CoA and PAPS, respectively, did not stimulate the activation of AAI in human cytosolic samples rich in these enzymes. These findings fully correspond to results obtained in our previous study utilising cytosolic samples of several human donors to identify the participation of cytosolic enzymes in AAI activation (Stiborova et al. 2003).

Conjugation reactions catalysed by phase II enzymes like SULTs have been shown to be involved in the metabolic activation of many carcinogenic nitroaromatics and aromatic amines (Arlt et al. 2002b, 2003, 2005; Dobbernack et al. 2011; Glatt and Meinl 2004; Glatt et al. 2016; Krais et al. 2016b). Hydroxylamine metabolites of nitroaromatics generally acquire increased reactivity upon sulfo-conjugation. We have shown in previous studies that the genotoxicity (i.e. DNA adduct formation) of another nitroaromatic, namely 3-NBA, is strongly influenced by human SULT1A1 and SULT1A2 (Arlt et al. 2002b, 2003, 2005). As expected treatment of *hSULT1A1/2* mice with 3-NBA resulted in higher 3-NBA-DNA adduct formation in several organs compared to WT mice, demonstrating that the expression of human SULT1A1/2 increases the activation of 3-NBA in vivo. These results confirm the suitability of the employed mouse model to study the role of human SULT1A enzymes in the metabolism of environmental toxins. Other studies have used this mouse line to demonstrate the involvement of human SULT1A1/2 in the activation of the food genotoxicants 2-amino-1-methyl-6-phenylimidazo[4,5-b]pyridine (PhIP) (Dobbernack et al. 2011), furfuryl alcohol (Sachse et al. 2014), 1-methylpyrene (Bendadani et al. 2014b) and methyleugenol (Herrmann et al. 2014).

In the case of AA, the *N*-hydroxyaristolactams may be further activated by *O*-acetylation or *O*-sulfo-conjugation to reactive esters capable of forming the same electrophilic species (aristolactam-nitrenium ions) as by nitroreductases and eventually the same AA-DNA adducts. However, such aristolactam-*N*-acetic acid esters or aristolactam-*N*-sulfuric acid esters have not yet been identified in in vivo studies on the metabolism of AA. When comparing DNA adduct formation of AAI and AAII between *hSULT1A1/2* and WT mice, we found no significant differences in adduct levels or adduct patterns in several organs examined. Expression of human SULT1A1/2 proteins detected by immunoblotting was found only in the *hSULT1A1/2* mice and not in WT mice. We also showed that the sulfotransferase activity in kidney and liver, the major organs involved in AA biotransformation, was many times higher in *hSULT1A1/2* than WT mice but the increase in sulfotransferase activity was not reflected in DNA adduct formation in these tissues as no differences were observed. These findings indicate that expression of human SULT1A1/2 does not contribute to the bioactivation of AAs in vivo.

Mouse models and cells derived from mice are often used to study experimental AAN and mechanisms related to AA carcinogenesis (Arlt et al. 2011; Baudoux et al. 2012; Krais et al. 2015; Nik-Zainal et al. 2015; Odell et al. 2013; Wang et al. 2012). Thus, it is also important to understand the role of murine Sults on AA bioactivation. Alongside the *Sult1a1(−/−)* mice we also included mice with a knockout of Sult1d1, an isoform that is preferentially expressed in mouse kidney (Alnouti and Klaassen 2006) and thus could contribute to the (geno)toxicity of AA in its target organ. In the tissues analysed adduct levels in *Sult1a1(−/−)* and *Sult1d1(−/−)* mice were similar to those in WT mice, except for AAI-treated livers of *Sult1d1(−/−)* mice where actually increased AAI-DNA adduct formation was observed. Collectively, these findings indicate that murine Sult1a1 and Sult1d1 do not contribute to the metabolic activation of AA in vivo. In contrast, we recently showed that Sult1a-mediated bioactivation of PhIP in the kidney depended on *Trp53* status in mice (Krais et al. 2016b) and other studies using the *Sult1a1(−/−)* and *Sult1d1(−/−)* mice mouse lines have demonstrated that expression of murine Sult1a1 and Sult1d1 can be a critical determinant in the genotoxicity of furfuryl alcohol (Sachse et al. 2014), methyleugenol (Herrmann et al. 2014) and 1-hydroxymethylpyrene (Bendadani et al. 2014a).

It should be mentioned that studies using in vitro systems (e.g. utilising recombinant human enzymes) to elucidate the role of human SULT1A1 in the bioactivation of AA have generated conflicting results (Martinek et al. 2011; Meinl et al. 2006; Sidorenko et al. 2014; Stiborova et al. 2011). For instance, whereas overexpression of human recombinant SULT1A1 in mammalian cells increased the genotoxicity (i.e. mutagenicity) of AA (Meinl et al. 2006), human recombinant SULT1A1 did not increase AA genotoxicity (i.e. DNA adduct formation) in a cell-free system (Stiborova et al. 2011). Although useful, these models have limitations, including the challenge of predicting the in vivo situation from reductionist in vitro results. In contrast, in vivo mouse models (i.e. *hSULT1A1/2* mice) take additional factors besides enzyme expression into account, such a route-of-administration, absorption, renal clearance and tissue-specific expression of XMEs. They also consider factors such as induction and/or inhibition of XMEs, as well as the presence of a variety of phase I and II enzymes, as critical determinants for the formation of reactive AA metabolites in vivo capable of forming DNA adducts. Several studies have shown that XMEs can behave differently in vitro and in vivo (Arlt et al. 2008, 2012; Krais et al. 2016a; Wohak et al. 2016). For example, some studies have revealed a paradox whereby CYP enzymes (particularly CYP1A1) appear to be more important for detoxification of benzo[*a*]pyrene in vivo, despite being involved in its metabolic activation in vitro (Arlt et al. 2008; Nebert et al. 2013).

Using ab initio calculations and molecular modelling have shown that the reaction free energy of dissociation of *N*-hydroxyaristolactam I is lower than that for *N*–OH-3-ABA (Stiborova et al. 2011, 2014b), the reactive intermediate of 3-NBA, the latter being used as a positive control in the present study. Hence, *N*-hydroxyaristolactam I decomposes spontaneously under the same conditions. Consequently, if the dissociation of the N–O bond in *N*-hydroxyaristolactam I (or its precursor, the open-ring hydroxylamine) is relatively fast, it is not the rate-limiting step in AAI-DNA adduct formation and any conjugation reaction (e.g. sulfation) would not make dissociation faster (i.e. would not lead to enhanced AAI-DNA adduct levels). These results indicate that the overall rate-controlling step during the reductive activation of AAI is not the enzymatic conjugation followed by spontaneous formation of the nitrenium/carbonium ion capable of reacting the DNA, but rather the initial nitroreduction mediated by NQO1 (Stiborova et al. 2011, 2014b). It should be noted that in the present study we did not treat the *hSULT1A1/2* mice with *N*-hydroxyaristolactams as these AA metabolites were not available to us. Although it might be useful to treat *hSULT1A1/2* mice with *N*-hydroxyaristolactams in future investigations, it is important to emphasise that humans are exposed to the natural mixture AA containing AAI and AAII and not *N*-hydroxyaristolactams 1 and 2. Interestingly, in vitro experiments with enzyme systems using AAI in the presence of NQO1, instead of *N*-hydroxyaristolactam I, showed that AAI-DNA adduct formation only increased after the addition of human SULT1B1 but not after the addition of SULT1A1 or SULT1A2 (Sidorenko et al. 2014), suggesting that human SULT1B1 may contribute to bioactivation of AA. Likewise, human SULT1B1 expressed in the target strain *Salmonella typhimurium* TA1538 enhanced the mutagenicity of AA, although somewhat less than expression of human SULT1A1 (Meinl et al. 2006). However, human SULT1B1 is primarily expressed in gut (Teubner et al. 2007); its expression in kidney is minimal (Meinl et al. 2006). Further studies are required to establish if human SULT1B1 contributes to the metabolic activation of AA in vivo.

In the present study, we investigated the contribution of the phase II enzyme SULT1A1 to the bioactivation of AAI and AAII in vivo using a transgenic mouse line carrying the functional human *SULT1A1*-*SULT1A2* gene cluster as well as *Sult1a1(−/−)* and *Sult1d1(−/−)* knockout mouse lines. In summary, our results indicate that conjugation by sulfation catalysed by SULT1A1/Sult1a1 does not play a role in the bioactivation of AAI and AAII in mice, whereas it is important in the activation of 3-NBA.
